# Cytokine Gene Expression in CD4 Positive Cells of the Japanese Pufferfish, *Takifugu rubripes*

**DOI:** 10.1371/journal.pone.0066364

**Published:** 2013-06-18

**Authors:** Tomoya Kono, Hiroki Korenaga

**Affiliations:** 1 Interdisciplinary Research Organization, University of Miyazaki, Miyazaki, Japan; 2 Interdisciplinary Graduate School of Agriculture and Engineering, University of Miyazaki, Miyazaki, Japan; Université Libre de Bruxelles, Belgium

## Abstract

CD4^+^ T (Th) cells are a central component of the adaptive immune response and are divided into distinct sets based on their specific cytokine production pattern. Several reports have suggested that fish possess Th subset activity similar to that of mammals. The aim of the present study was to isolate CD4^+^ T cells from the blood of Japanese pufferfish, *Fugu rubripes*, and to characterize their cytokine expression profile. We produced a specific antibody against *Fugu* CD4 and performed cell sorting with the magnetic activated cell sorting system. Sorted *Fugu* CD4^+^ cells were characterized by morphology and expression analysis of cell marker genes. *Fugu* CD4^+^ cells expressed T-cell marker genes but not macrophage or B-cell marker genes. In addition, peripheral blood lymphocytes were stimulated with lipopolysaccharide (LPS), polycytidylic acid (polyI:C), concanavalin A (ConA) prior to sorting, and then Multiplex RT-PCR was used to examine the expression of Th cytokines by the stimulated *Fugu* CD4^+^ cells. LPS and polyI:C stimulation upregulated the expression of Th1, Th17 and Treg cytokines and downregulated the expression of Th2 cytokines. ConA stimulation upregulated the expression of all Th cytokines. These results suggest that fish exhibit the same upregulation of Th-specific cytokine expression as in mammals.

## Introduction

Immune responses are greatly dependent on the induction of helper T (Th) cells during the initial exposure to antigen through the T-cell receptor (TCR) [1]. Th cells is specified with several cell-surface markers in the mammals. Especially, CD4 is recognized as one of the most effective surface markers for identifying Th cells subset [2]. In humans, CD4 is weekly in myeloid cells such as monocyte, dendritic cells, and neutrophils [3], [4], [5]. It is reported that CD4^+^ myeloid cells is hypothesized to amplify T-cells and various innate immune cells with Fcγ receptor to enhance responses, however the mechanism was not really cleared compared with CD4^+^ T-cells [6]. CD4^+^ Th cells can be classified into different subsets based on their cytokine profile. Thl, Th2, Th17 and regulatory T cells (Treg) are Th subsets that can be functionally distinguished by the production of specific cytokines such as IFN-γ, IL-4, IL-17 and TGF-β1, respectively [7], [8]. Through the production of these distinct sets of cytokines and other soluble and cell-bound products, such as antimicrobial peptides, complement fragments, cytokines, and chemokines, Th subsets may act as immune effectors that eliminate infected cells [9], [10]. The helper T cell paradigm has been confirmed in mice and humans [11]. Recently, it has been determined that Th cells possess greater heterogeneity and plasticity than previously thought [12]. Other Th subsets including Th3, Th9, Th22, Tr1 and Tfh cells have been identified, but it is unclear if these subsets are distinct from the four known lineages [13]. Studies of Th subsets in non-mammalian species have been greatly limited by the lack of specific antibodies to detect the CD4 protein [14]. There have been only two previous reports on the isolation and analysis of CD4^+^ cells in fish by using specific antibodies to CD4 [15], [16].

Over the past decade, within teleost fish, a number of fish cytokine genes have been discovered by PCR-based homology cloning with degenerate primers and *in silico* searches of available genomic databases. The *in silico* discoveries were achieved after the release of genome databases by exploring the chromosomal synteny between the mammalian and fish genomes [17]. The following IL cytokines have been isolated in fish: IL-1, -2, -4, -6, -7, -8, -10, -11, -12, -13, -15, -16, -17, -18, -19, -20, -21, -22, -23, -26 and -34 [17], [18], [19], [20], [21], [22], [23]. In addition, two fish tumor necrosis factor (TNF) super family genes (TNF-α and TNF-N) [24], interferon (IFN) family genes (type-I IFN and IFN-γ) [25], [26], and many chemokine family genes [27] have been identified in fish species. The potent regulatory cytokine transforming growth factor-β1 (TGF-β1) has also been described [28]. Although the isolation of fish cytokines has progressed well, there are few reports regarding their function, and the mechanism of the regulation of Th differentiation by cytokines has not been elucidated.

In teleost fish, two types of CD4 molecules, CD4 (refered to as CD4-1) and CD4REL (referred to as CD4L, or CD4-2), have been identified from several fish species including Japanese pufferfish *Takifugu rubripes* (*Fugu*) [29], rainbow trout *Oncorhynchus mykiss* [30], common carp *Cyprinus carpio* [31], channel catfish *Ictalurus punctatus* [32], Atlantic halibut *Hippoglossus hippoglossus* [33] and sea bass *Dicentrarchus labrax* [34]. In mammals, CD4 is composed of four extracellular immunoglobulin (Ig)-like domains, a transmembrane region and a cytoplasmic tail [35], [36]. This cytoplasmic tail, which contains a conserved binding site, interacts with the protein tyrosine kinase p56^lck^ to induce the activation of T cells. Similar to known mammalian CD4 molecules, teleost CD4 is also predicted to contain four extracellular Ig-like domains, a transmembrane region, and a cytoplasmic tail in which the p56^lck^ domain is conserved in the teleost species. However, it is reported that teleost CD4REL is possibly composed of only two or three extracellular Ig-domains [30]. All of the teleost CD4 molecules (CD4 and CD4REL) identified have a p56^lck^ binding motif in their cytoplasmic domain. Furthermore, the expression analysis of *CD4* genes in several fish species has suggested that teleost CD4^+^ cells may function as helper T cells similar to mammalian CD4^+^ cells, despite low sequence identities to mammalian *CD4* genes [31], [34], [37]. In green spotted pufferfish, *Tetraodon nigroviridis*, CD4REL expression is associated with teleost Treg-like cells [16]. Toda *et al*. [15] suggested that CD4^+^ T cells in ginbuna carp, *Carassius auratus langsdorfii*, are equivalent to helper T cells like mammals. However, detailed investigations of CD4 molecules and CD4^+^ T cells in fish have not yet been conducted.

Little is known about the repertoire of cytokines produced by teleost CD4^+^ T cell subsets, and in the case of immune stimulation, no information is currently available. The Japanese pufferfish *Takifugu rubripes* has advantages over other fish species for use in immunology studies. Gene identification is easier in a diploid fish such as *Fugu* than in a tetraploid fish [38]. Several immune-related genes including Th cytokines have been identified in *Fugu* [39]. Moreover, the body size of *Fugu* is larger compared with the other model fish species such as medaka *Oryzias latipes* and zebrafish *Danio rerio*, making it easier to culture tissue cells [40]. Therefore, we selected *Fugu* for our study of CD4^+^ T-cell subsets in fish. We sorted CD4^+^ cells from the blood of the Japanese pufferfish *Fugu* by using an antiserum produced against *Fugu* CD4, and we investigated the expression of Th cytokine genes in the sorted CD4^+^ T cells by a multiplex RT-PCR assay. This report extensively examines the immune responses of CD4^+^ T cells in a teleost fish and provides important information regarding the phylogeny of immune responses.

## Materials and Methods

### Fish

Japanese pufferfish (*Fugu*) *Takifugu rubripes* (mean weight, 200 g) were obtained from Matsumoto Fisheries Farm, Miyazaki, Japan. Prior to their use in the study, the fish were first acclimatized in an aerated seawater tank at 20°C and fed a commercial diet (Sango, Higashimaru Co., Ltd., Kagoshima, Japan) at 1% body weight per day for two weeks under a natural photoperiod. All experiments were conducted in accordance with the guidelines for the care and use of laboratory animals at the University of Miyazaki.

### Production of Anti–*Fugu* CD4 Polyclonal Antibody and Confirmation of Its Specificity

#### Production of the *Fugu* CD4 polyclonal Ab

The peptide NKVIKHENWDTALSD of the *Fugu* CD4 (Acc. No. NP_001072091) extracellular domain was synthesized and conjugated to keyhole limpet hemocyanin as a carrier protein. Rabbits were injected with the conjugated peptide, followed by multiple boosts. The serum was obtained 49 days after the initial injection and stored at −20°C. The peptides and antibody (Ab) were prepared by Operon Biotechnology (Tokyo, Japan). In this study, the serum was used as anti-*Fugu* CD4 Ab.

Reactivity of anti-*Fugu* CD4 Ab to the synthesized CD4 peptide was assessed by enzyme-linked immunosorbent assay (ELISA). Synthesized CD4 peptide (5 µg/ml) in phosphate-buffered saline (PBS) [10 mM sodium phosphate and 150 mM NaCl (pH 7.2)] was added to coating plates Immunoplate Maxisorp F96 (Nalge Nunc International, Rochester, NY, USA) and kept overnight at 4°C. After washing the plates with PBS containing 0.2% tween, diluted anti-*Fugu* CD4 Ab (1/1,000∼1/128,000) was added and incubated for 2 h at room temperature. After washing with PBS containing 0.2% tween, the amount of bound Ab to CD4 peptide was detected by incubation with horseradish peroxidase (HRP)–conjugated goat anti-rabbit IgG (Sigma-Aldrich, St Louis, MO, USA). After the plate was washed with PBS containing 0.2% tween, color development was conducted by adding o-Phenylenediamine dihydrochloride substrate (0.4 mg/ml) (Sigma). Optical density (O.D.) was measured at 492 nm with a Multiskan FC microplate reader (Thermo Fisher Scientific, Pittsburgh, PA, USA).

### The Establishment of CHO Cells Expressing *Fugu* CD4 (*Fugu* CD4^+^-CHO)

Full-length *Fugu* CD4 cDNA was amplified by PCR with forward primer 5′- ATGACCTTCGTCAGCAGACA-3′ and reverse primer 5′- CGTCCTGTAGAAGCCTTTAG-3′ and cloned into the pGEM-T vector (Promega, Madison, WI, USA). To obtain *Fugu* CD4 cDNA with an N-terminal Kozak sequence (CCACC), plasmid cDNA was PCR-amplified. The PCR conditions were as follows: one cycle of 94°C for 3 min, 30 cycles of 94°C for 30 s, 60°C for 30 s and 72°C for 45 s, followed by one cycle of 72°C for 5 min. PCR products were separated on 1.5% agarose gels and visualized by staining the gels in 1x TBE [100 mm Tris, 100 mm boric acid, 2 mm EDTA (pH 8.0)] containing 100 ng/ml ethidium bromide (Sigma-Aldrich). PCR products were then ligated into the pEBMulti vector (Wako, Osaka, Japan) following the manufacturer’s instructions. pEBMulti vector is distributed to daughter cells by an episomal replicating system. Following transfection into *Escherichia coli* TAM competent cells (Active Motif, Carlsbad, CA, USA), recombinants were identified by red–white color selection when grown on MacConkey agar (Sigma-Aldrich). Plasmid DNA from at least three independent clones was extracted by using the QIAprep Spin Miniprep Kit (QIAGEN, Hilden, Germany) and sequenced by using an ABI 377 Automated Sequencer (Applied Biosystems, Foster City, CA, USA).

Chinese hamster ovary (CHO) cells were cultured in α-minimum essential medium (α-MEM; Invitrogen, Carlsbad, CA, USA) supplemented with 10% fetal calf serum (GIBCO, Grand Island, NY, USA) and 1% penicillin-streptomycin (GIBCO) at 37°C and 5% CO_2_. Cells were transiently transfected by using X-tremeGENE 9 DNA Transfection Reagent (Hoffmann-La Roche Inc., Basle, Switzerland) according to the manufacturer’s instructions. Cells were plated one day before transfection at 1×10^6^ cells, transfected with 5 µg constructed plasmid, and selected by using growth medium containing 0.5 mg/ml of G418 (Invitrogen).

### Morphological Analysis of *Fugu* CD4^+^-CHO Cells

Following the selection with G418, *Fugu* CD4^+^-CHO cells (1×10^6^ cells) were incubated with anti-*Fugu* CD4 Ab (1∶500 dilution) at 4°C for 30 min. After washing two times with PBS, the cells were incubated at 4°C for 20 min with goat anti-rabbit IgG (PE) Ab (Abcam, Cambridge, MA, USA) diluted 1∶500 in PBS (1% BSA). After washing twice with PBS, the cells were suspended in 100 µl PBS. Cytospin preparations were performed by centrifugation at 1,000****rpm for 2 min in a StatSpin Cytofuge 2 centrifuge (StatSpin Technologies, Norwood, MA, USA). Prepared cytospin slides were stained with 4′ 6-diamidino-2-phenylindole (DAPI) (300 nM in PBS; Sigma). This diluted DAPI staining solution was added to the coverslip preparation, making certain that the cells were completely covered, and then cells were incubated for 1 min. After washing two times with PBS, the fluorescence of the cells was examined under an Axiovert 40CFL ﬂuorescence microscope (Carl Zeiss, Oberkochen, Germany).

### Flow Cytometry (FCM) Analysis of *Fugu* CD4^+^-CHO

As described in the previous paragraph, anti-*Fugu* CD4 Ab and goat anti-rabbit IgG (PE) Ab were incubated with *Fugu* CD4^+^-CHO cells. As a negative control, *Fugu* CD4^+^-CHO cells were incubated with rabbit normal Ab as the primary Ab and anti-rabbit IgG PE Ab (Abcam) as the secondary Ab. Fluorescence analysis was performed by using an EPICS XL flow cytometer (Beckman Coulter, Brea, CA, USA) and FlowJo software (Tree Star, Inc., San Carlos, CA, USA ). Histograms of cell number versus fluorescence intensity were recorded for at least 10,000 cells per sample. The setting of negative gate was used with the only secondary antibody reaction to the cells analysis.

### Verification of Specificity of Anti-*Fugu* CD4 Ab by Western Blotting

#### Synthesis of recombinant *Fugu* CD4 protein in an Sf21 insect cell-free protein synthesis system

*Fugu* recombinant CD4 protein (*Fugu* rCD4) was synthesized by using an Sf21 insect cell-free protein synthesis kit (Shimadzu, Kyoto, Japan). The *Fugu CD4* gene was amplified by PCR (forward primer, ATGACCTTCGTCAGCAGACAC; reverse primer, GGGGTACCCGTCCTGTAGAAGCCTTTAGG) and the product was ligated into the pTD1-G8-FLAG vector (Shimadzu). Following transformation into *E. coli* TAM competent cells (Active Motif), recombinants were identified via ampicillin selection when grown on LB agar (Sigma-Aldrich). Plasmid DNA was then purified by using a QIAprep Spin miniprep kit (Qiagen). For efficient transcription, the plasmid cDNA was linearized by PCR (forward primer, GCAGATTGTACTGAGAGTG; reverse primer, GGAAACAGCTATGACCATG). The linearized cDNA was transcribed by using the T7 RiboMAX Expression Large Scale RNA Production System (Promega) and was purified with NICK columns (GE Healthcare, Buckinghamshire, UK). Cell-free protein synthesis was performed on a 500 µL scale by using the Transdirect insect cell (Shimadzu) and Promega FluoroTect Green_Lys_ *in vitro* Translation Labeling System (Promega). Reactions were performed by adding prepared mRNA followed by incubation at 25°C for 4 h. The recombinant *Fugu* CD4 proteins suspended in SDS-PAGE loading buffer (Bio-Rad Laboratories, Hercules, CA, USA) were heated for 5 min at 95°C and loaded onto a 15% precast e-PAGEL gel (ATTO, Tokyo, Japan). After electrophoresis, the fluorescent-labeled *Fugu* rCD4 was detected by a Typhoon FLA 9000 laser scanner (GE Healthcare). The synthesized *Fugu* rCD4 was stored at −20°C until further use for Western blotting.

#### Preparation of cell lysates

*Fugu* CD4^+^-CHO cells (1×10^7^ cells) and *Fugu* PBLs (1×10^6^ cells) were lysed in 400 µl of modified RIPA buffer (10 mM Tris-HCl, pH 7.2, 150 mM NaCl, 1 mM EDTA, 1% Triton X-100, 1% deoxycholate, 0.1% SDS, 1% aprotinin, 1× Protease Inhibitor Cocktail;Sigma, USA) on ice for 30 min. The lysate was centrifuged at 10,000×g for 15 min, and the pellet was discarded. The supernatant was stored at −20°C until further use for Western blotting.

#### Specificity of anti-*Fugu* CD4 Ab by western blotting

The *Fugu* rCD4 and lysates of *Fugu* CD4^+^-CHO and *Fugu* PBLs were suspended in SDS-PAGE loading buffer (Bio-Rad Laboratories), heated for 5 min at 95°C and loaded onto a 15% precast-PAGEL gel (ATTO). After electrophoresis, proteins were transferred onto a nitrocellulose membrane (Invitrogen). The membrane was blocked with Western breeze blocking solution for 30 min on a rotary shaker at slow speed. After rinsing with ultra-filtered water, the membrane was incubated with the primary Ab, rabbit anti-*Fugu* CD4 Ab (1∶1,000 dilution), for 1 h and rinsed again with Ab washing solution and ultra-filtered water. The washed membrane was incubated in secondary anti-rabbit-Ig/AP Ab solution (1∶2,000 dilution; Invitrogen) for 30 min. The membrane was again washed extensively and then incubated in chromogenic substrate solution (5-bromo-4-chloro-3-indolyl-phosphate/nitro blue tetrazolium) until purple bands developed.

### Isolation of *Fugu* CD4 cells by Magnetic Activated Cell Sorting (MACS)

#### T-cell purification from peripheral blood leukocytes (PBLs)

Blood was collected in heparinized syringes from three fish and diluted 1∶4 in Hanks’ Balanced Salt Solution (HBSS). PBLs were isolated by Percoll (GE Healthcare) density gradient (1.065 and 1.050 g/ml) centrifugation at 1,500 rpm for 50 min. After centrifugation, PBLs were collected, washed three times with HBSS and suspended in RPMI 1640 (Invitrogen) supplemented with 5% FBS and 1% penicillin/streptomycin (Invitrogen). To purify the lymphocytes, we used the method described by Sugamata et al [40]. PBLs were incubated for 1 h in a ﬂask (TPP Techno Plastic Products, Trasadingen, Switzerland) pretreated with heat-inactivated *Fugu* serum. Following incubation, the lymphocyte-enriched supernatant in the ﬂask was carefully collected to purify B cells and T cells. The lymphocyte-enriched supernatant was filtered with nylon fiber columns (Polysciences, Warrington, PA, USA) to remove B cells. The through fraction from the nylon mesh column was pooled as a purified T cell population. Adhered cells were washed thoroughly several times with RPMI 1640 medium to remove neutrophils, which show less adhesive properties than monocytes. Following the washes, adherent monocytes were incubated with 5% FBS RPMI 1640 and finally collected from the plastic surface.

### Morphology and Cell Marker Analysis in the Purification Process of *Fugu* T Cells

PBLs, monocytes, lymphocytes and T cells were stained with May-Grünwald-Giemsa and pictures were taken under an Axiovert 40CFL microscope (×400; Carl Zeiss). The morphology of cell types recognized by *Fugu* CD4 was also examined under an Axiovert 40CFL ﬂuorescence microscope (Carl Zeiss). Anti-*Fugu* CD4 Ab and goat anti-rabbit IgG (PE) Ab were reacted with PBLs, monocytes, lymphocytes and T cells, and DAPI staining was performed as described in a previous section (*Morphology analysis of CHO cells*). Fluorescence of cells was examined under an Axiovert 40CFL ﬂuorescence microscope (Carl Zeiss). We also analyzed the amount of CD4^+^ cells in PBLs, monocytes, lymphocytes and T cells by FCM as described in a previous section (*FCM analysis*).

For expression analysis of cell marker genes, total RNA of PBLs, monocytes, lymphocytes and T cells was extracted and reverse transcribed into cDNA. Briefly, total RNA was extracted from individual cells by using ISOGEN (Nippon Gene, Tokyo, Japan) according to the manufacturer’s instructions. After extraction, RNA was treated with recombinant DNAse I (Takara Bio, Shiga, Japan) according to the manufacturer’s instructions to digest contaminating genomic DNA. Total RNA concentration was determined at 260 nm (NanoDrop-1000, Thermo Fisher Scientific, Waltham, MA) and RNA purity was verified by evaluating the ratio of the optical density at 260 nm vs the optical density at 280 nm. Total RNA was diluted to 0.2 µg/µl in nuclease-free water. Reverse transcription was performed by using the cDNA Reverse Transcription kit (Toyobo, Japan) following the manufacturer’s protocol, and the cDNA was stored at −20°C.

RT-PCR with gene-specific primers (Table 1) was performed by using cDNA prepared as described above (see cDNA production). Primers for *Fugu* β-actin (Table 1) were used as an internal control for RT-PCR. PCR conditions were described in a previous section (*The establishment of CHO cells*). The cycle number was 25 cycles for β-actin and 35 cycles for the marker genes.

**Table 1:** Primers designed for expression analysis of marker genes in this study.

Name	Sequence (5′–3′)	Length (mer)
*CSF1R1* Fw	TTTACCGACACCGCGGGATT	20
*CSF1R1* Rv	GCCGCTGTCACTTCTAATGTA	20
*CSF1R2* Fw	GGCATGAACGTGACTGTTGA	20
*CSF1R2* Rv	CATGGTACCCAAGGTGACTT	20
*CD4* Fw	AAGCCTCAGAGGGAACAGAA	20
*CD4* Rv	GAAGAACGTGGTCGATACGA	20
*CD4REL* Fw	GGAAGCAGATGCAGGAATGT	20
*CD4REL* Rv	GCCTGTGACTCTGATATCCA	20
*CD8α* Fw	CCCAGGTGGACATTCATTGT	20
*CD8α* Rv	TTGTTGCCTCGGCGTCGTTT	20
*TCRα* Fw	AGCGCATGTCTGGCCACAGGTTTCA	25
*TCRα* Rv	GACTGATACGCAGACGAAGAGTCATCAGG	29
*TCRβ* Fw	TCCTCCAGAGAGTGTCGCAA	20
*TCRβ* Rv	TGCAGCTTCCAGGCCAGAAA	20
*TCRγ* Fw	CAGGGCTGGTTGTCATAGAA	20
*TCRγ* Rv	GCATCTGAGACGACGAGTCT	20
*TCRδ* Fw	TCAGCAGCAGAGAAGGAGAA	20
*TCRδ* Rv	ACTCAGGACTGTCGGATCTT	20
*CD3ε* Fw	CCAACCGATTAGAGCGAATCAGAGGC	26
*CD3ε* Rv	CCTGATTCCTTCCCGGATCCAGC	23
*CD28* Fw	GAAGGTAGAAGGGAAGACAAGCGTCCC	27
*CD28* Rv	CTTTCACATAGCAGGACAGGGCGAC	25
*CD154* Fw	CTGGGACTGGAAACATGGTCACTCTCGG	28
*CD154* Rv	GCAACACTGTACAGGGTTCCATCCGCGG	28
*IgL* Fw	GAAGGTAGAAGGGAAGACAAGCGTCCC	27
*IgL* Rv	CTTTCACATAGCAGGACAGGGCGAC	25
*IgM* Fw	CTGGGACTGGAAACATGGTCACTCTCGG	28
*IgM* Rv	GCAACACTGTACAGGGTTCCATCCGCGG	28
*T-bet* Fw	CCGTGACAATTACGACACGC	20
*T-bet* Rv	GAGAGGTAGCCTTGGGGGTA	20
*GATA-3* Fw	CAACCGACCTCTGACCATGA	20
*GATA-3* Rv	CCATGCTGTCCTGGGACTTT	20
*FoxP3* Fw	GTGGAAGGTCCAACAGGACA	20
*FoxP3 Rv*	CGGAATCTGACTGCTGGTCT	20

### Isolation of CD4^+^ Cells from Purified T cells by MACS

The isolated T cells (1×10^6^ cells) from PBLs were diluted in 90 µl PBS and were marked with 10 µl anti-*Fugu* CD4 Ab (diluted 1∶100) at 4°C for 30 min. Unbound antibodies were removed by centrifugation at 300x G for 5 min at 4°C. The cells were resuspended in 90 µl MACS buffer (PBS, 0.5% BSA, and 2 mM EDTA; Miltenyi Biotec, Bergisch Gladbach, Germany). Then, the cells were incubated with 10 µl of goat anti-rabbit IgG bound magnetic microbeads (Miltenyi Biotec) (diluted 1∶500 in MACS buffer) at 4°C for 15 min. After washing twice with MACS buffer, the cells were resuspended with 500 µl of MACS buffer and applied to the MACS MS columns (Miltenyi Biotec). The cells that passed through the column were washed four times with 500 µl of MACS buffer. The column was removed from the separator, and the CD4^+^ cells were eluted by using the plunger. Next, CD4^+^ cells were incubated at 4°C for 20 min with 100 µl anti-rabbit IgG PE Ab (Abcam, USA) diluted 1∶1000 in PBS (1% BSA). After washing twice with PBS, the cells were resuspended in PBS, and FCM was performed as described above (*Flow cytometry (FCM) analysis* and *Morphology and cell marker analysis*). Expression analysis of cell marker genes was conducted by quantitative real-time PCR. The assay was performed in a 20 µL reaction system containing 1×THUNDERBIRD SYBR qPCR Mix, 1×ROX reference dye (Toyobo), 0.5 µM primers of specific genes (Table 1) and 50 ng cDNA from three individuals. Thermal cycler parameters on ABI 7300 (Applied Biosystems) include 95°C for 3 min, 40 cycles of denaturation at 95°C for 15 s, annealing and extension at 60°C for 1 min. After 40 cycles, the PCR products were analyzed using the heat dissociation protocol to confirm that every single PCR product was detected by SYBR green dye. Quantitative values were obtained from the threshold PCR cycle number (Ct) at which the increase in signal associated with an exponential growth for PCR product started to be detected three times. The normalization of relative expression was calculated by the comparative Ct method (2^−ΔΔCt^ method) with *β-actin* as a housekeeping gene. All data are given as mean ± S.D.

### Expression Profile of Cytokine Genes in CD4^+^ cells under Immune-stimulated Conditions

#### *In vitro* stimulation with LPS, polyI:C and ConA

The isolation of leukocytes from blood of three different individuals was described in a previous section (*T-cell purification*). The cell number was adjusted to 1×10^7^ cells/ml, and the cells were stimulated with bacterial lipopolysaccharide (LPS; Sigma; 20 µg/ml), polyinosinic:polycytidylic acid (polyI:C; Sigma; 20 µg/ml) or Concanavalin A (ConA; 20 µg/ml) for 0, 6, 12 and 24 h at 25°C in RPMI 1640 medium (Invitrogen) supplemented with 5% FBS and 1% streptomycin/penicillin (Invitrogen). A control group was cultured with HBSS except stimulant for same time course. These stimulation was conducted with three different individuals. After stimulation, *Fugu* CD4^+^ cells were isolated from each of the treated PBL groups as described above (*Isolation of Fugu CD4 cells*).

### Multiplex RT-PCR of Isolated *Fugu* CD4^+^ Cells

The cytokine multiplex analysis was conducted by using the multiple assay panel as reported previously [41]. In the present study, cytokines produced by Th cells (including cytokines related to the differentiation of Th cells) were targeted. Target cytokine genes were Th1 cytokines including IFN-γ, IL-2, and TNF-α, Th2 cytokines including IL-4/13-A and -B, Th17 cytokines including IL-17A/F-3, and Treg cytokines including TGF-β1 and IL-10. Total RNA extraction from the sorted *Fugu* CD4 cells and DNase I treatment were performed as described above (*Morphology and cell marker analysis*). Reverse transcription to cDNA was conducted by using reverse transcriptase, RNase inhibitor, Kan^r^ RNA and 1× RT Master Mix Buffer supplied in the GenomeLab GeXP Start Kit (Beckman Coulter) according to the manufacturer’s recommendations. After reverse transcription, PCR was performed with each reaction containing 9.3 µL reaction mixture, 0.02 µM forward primer set mix, 5 mM MgCl_2_, 3.5 U Thermo Start *Taq* DNA polymerase (Thermo Fisher Scientific), and 1 × PCR Master Mix Buffer (GenomeLab GeXP Start Kit; Beckman Coulter) containing 10 mM HCl, 50 mM KCl, 0.3 mM of each dNTP, 0.02 µM Kan^r^ gene PCR forward primer, 1 µM universal reverse primer, and 1 µM D4-labeled universal forward primer. Amplicons from multiplex RT-PCR were diluted 1∶100 in distilled water, and then 2 µL of the diluted sample was added to 37.75 µL sample loading solution along with 0.25 µL of DNA size standard-400 (GenomeLab GeXP Start Kit; Beckman Coulter). The GeXP Genetic Analysis system matched each PCR product based on size by capillary gel electrophoresis (CEQ8000 Automated Sequencer; Beckman Coulter) with the appropriate gene and measured the dye signal strength in arbitrary units of optical fluorescence, defined as the fluorescent signal minus background. Next, the data was normalized to kanamycin by using the GeXP profiler software (eXpress Analysis) with the area under the curve set to 1. This step minimizes intercapillary variation. The expression level of each cytokine gene was calculated by normalization of internal control genes (β-actin [42] and GAPDH [43]) by using GeXP Quant Tool. The assay was carried out in triplicate for each three individuals and then pooled the results.

### Statistical Analysis

The statistical significance of differences between stimulated tissue and normal tissue was determined by using a paired-sample *t*-test because the expression data consisted of sets of samples from individual fish. Differences were considered to be significant when *P*<0.01.

## Results

### Specificity of Anti-*Fugu* CD4 Ab

The results of ELISA revealed that pre-immune rabbit serum (normal serum) did not recognize the synthesized *Fugu* CD4 peptide, but after immunization rabbit antiserum (anti-*Fugu* CD4 Ab) possessed high titers of antibody against the *Fugu* CD4 peptide. The optical densities at 492 nm were plotted against the dilution of the anti-*Fugu* CD4 Ab or normal serum (Fig. S1), where ∼1/16,000 diluted anti-*Fugu* CD4 Ab showed a high ELISA signal ratio (O.D. 3.0 at 492 nm).

**Figure 1:**
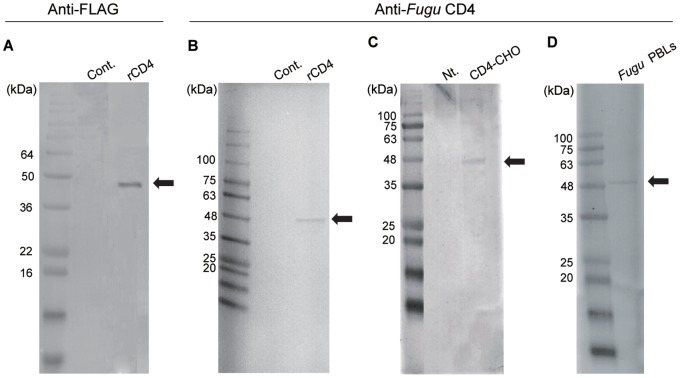
The confirmation of antibody specificity using Western blotting. The recombinant CD4 was detected with A) anti-FLAG Ab and B) anti-*Fugu* CD4 Ab. C) *Fugu* CD4-CHO cells and D) *Fugu* PBLs lysates were detected with anti-*Fugu* CD4 Ab. The position of molecular weight markers is indicated to the left of each Western blot. The arrow indicates the predicted size of *Fugu* CD4 protein. Cont, translated with control plasmid. Nt, the lysate of non-transfected CHO.

In Western blot analysis, a strong band for *Fugu* CD4 was only observed when anti-FLAG Ab or anti-*Fugu* CD4 Ab was reacted with FLAG-tagged recombinant *Fugu* CD4 synthesized in an insect cell-free protein synthesis system (Fig. 1A, B). The size of the bands corresponded to the estimated size of the *Fugu* CD4 construct (approx. 50 kDa). Anti-*Fugu* CD4 Ab also weakly detected the same sized protein in lysates of *Fugu* CD4^+^-CHO cells and *Fugu* PBLs (Fig. 1C, D).

**Figure 2:**
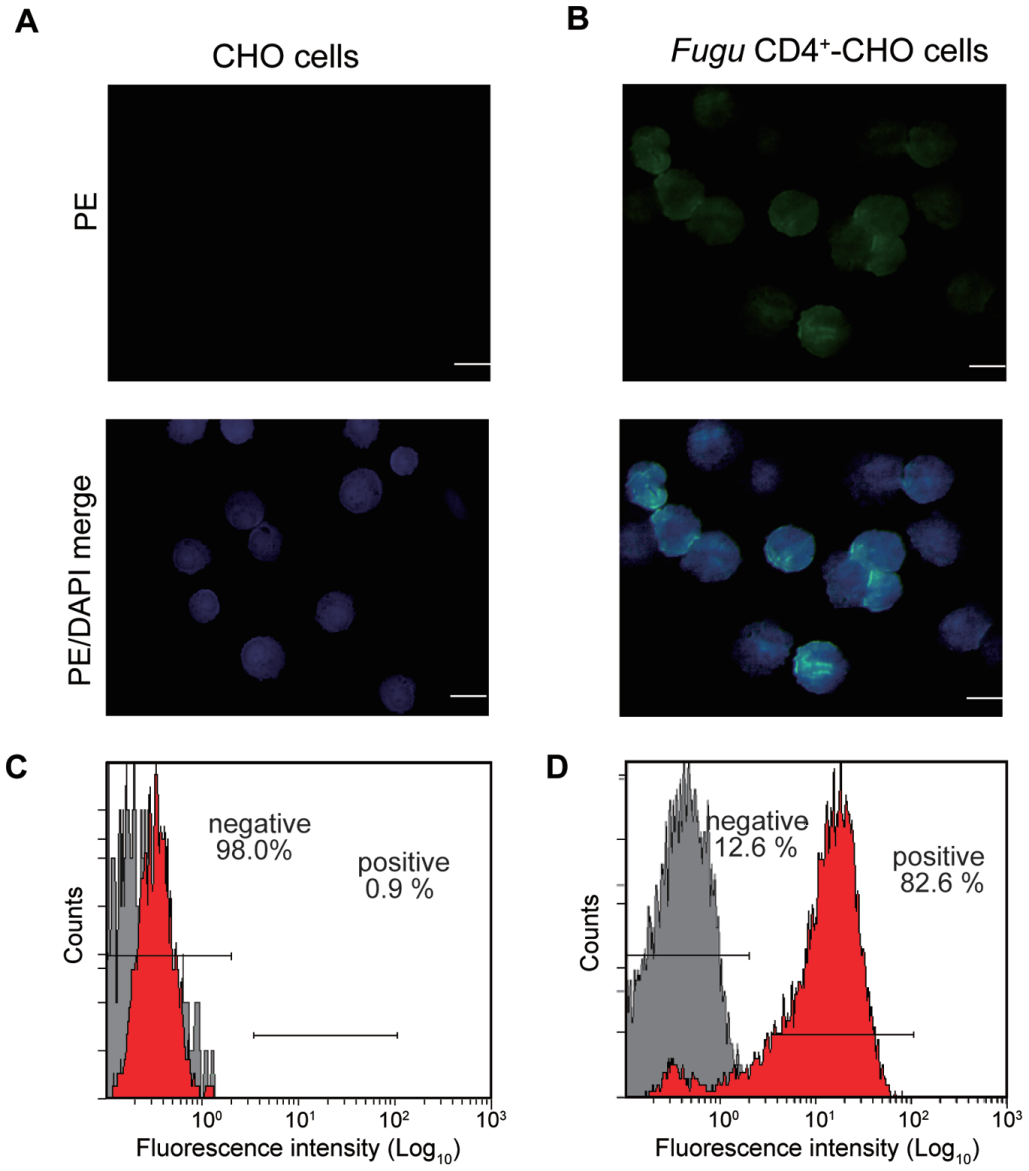
Reactivity of anti-*Fugu* CD4 Ab to *Fugu* CD4^+^-CHO cells. Immunofluorescence of A) control CHO cells and B) *Fugu* CD4^+^-CHO cells. Cells were incubated with anti-*Fugu* CD4 Ab as primary Ab and goat anti-rabbit IgG (PE) as secondary Ab and DAPI to mark cell nuclei. Scale bar equals 20 µm. Stained cells C) control CHO cells and D) *Fugu* CD4^+^-CHO cells were also analyzed by flow cytometry. The setting of negative gate was used with the only secondary antibody reaction to the cells analysis (gray peak).

Immunofluorescence staining of *Fugu* CD4^+^-CHO cells with anti-*Fugu* CD4 Ab, followed by PE-conjugated secondary antibodies, showed that CD4 was located on the surface of cells (Fig. 2B), while untransfected CHO cells (control) did not show any PE staining (Fig. 2A). Furthermore, flow cytometry (FCM) analysis confirmed the expression of *Fugu* CD4 on the cell surface of *Fugu* CD4^+^-CHO cells by using anti-*Fugu* CD4 Ab. The antibody reacted to 82.6±2.5% of *Fugu* CD4^+^-CHO cells and only 0.9±0.2% of untransfected CHO cells (Fig. 2C, D).

### Purification of *Fugu* Peripheral Blood T cells

**Figure 3:**
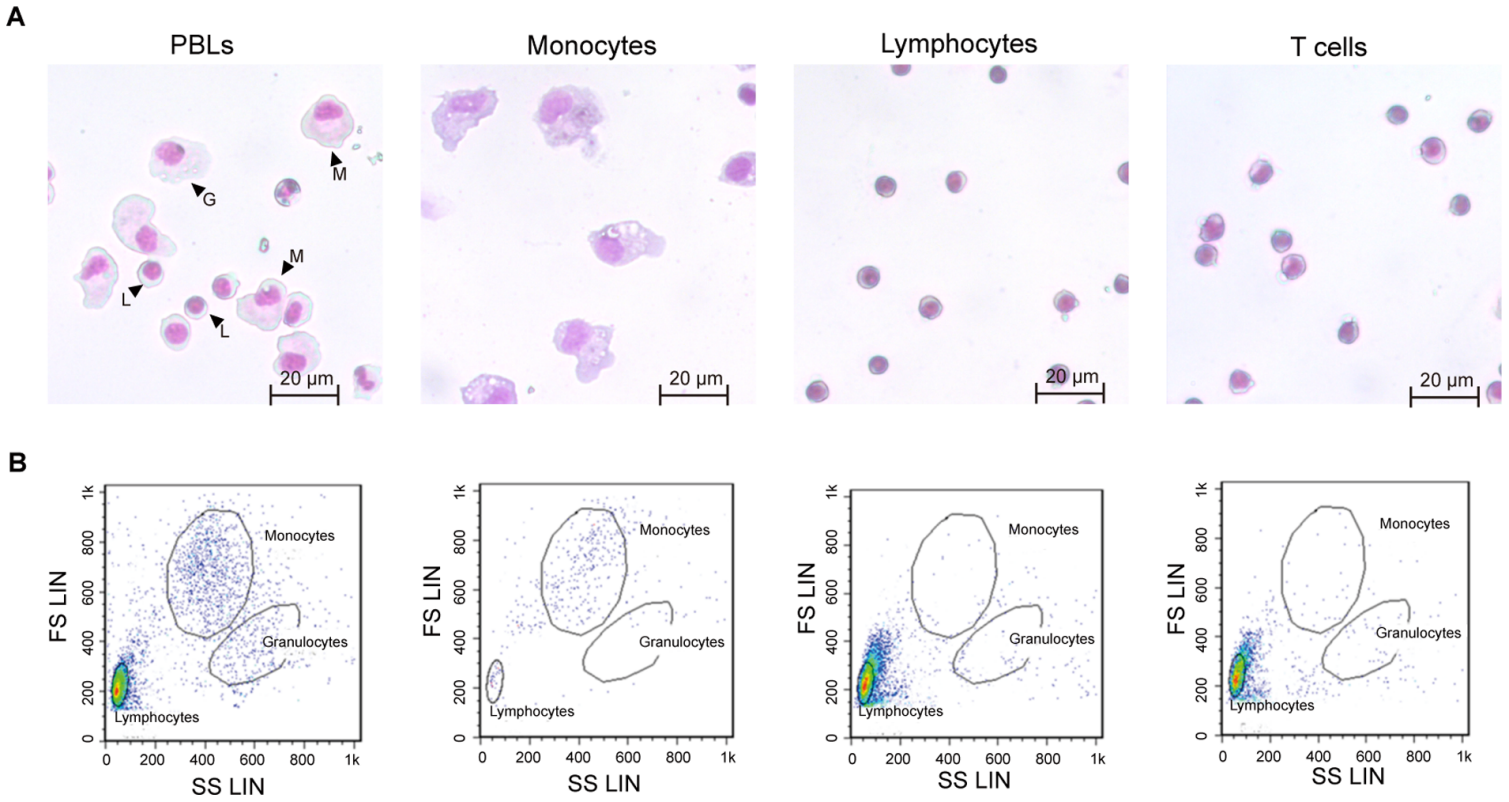
Discrimination of *Fugu* PBLs, monocytes, lymphocytes and T cells. A) The morphology of purified cells was examined by microscope after May-Grünwald-Giemsa staining. G, L and M indicate granulocytes, lymphocytes and monocytes, respectively. B) Scattergram of the flow cytometric profile of purified cells. Different cell subpopulations were identified on the basis of their size and complexity, and cellular debris was excluded. Three populations (granulocytes, monocytes and lymphocytes) in PBLs were isolated by analytical gates. The monocyte, lymphocyte and T cell populations were mostly pure, and contamination with other populations was low.

To efficiently isolate CD4^+^ cells, we purified T cells from PBLs by using density gradient centrifugation and the adherent property of monocytes and neutrophils on plastic surfaces. During the process of T-cell purification, cells were stained with May–Grünwald–Giemsa dye and microscopically examined. The lymphocyte, monocyte and granulocyte populations were highly enriched from PBLs. The monocytes are large, round cells with abundant cytoplasm often containing vacuoles. The lymphocytes and T cells had a small size with sparse cytoplasm and quite similar morphology. The each purified cells was not observed the contamination with other population (Fig. 3A). In the following FCM analysis, forward/side scatter analysis indicated the presence of three populations, gated as lymphocytes, monocytes and granulocytes (Fig. 3B). With respect to the purity of lymphocytes and T-cells, these populations are commonly observed in the lymphocytes gate.

**Figure 4:**
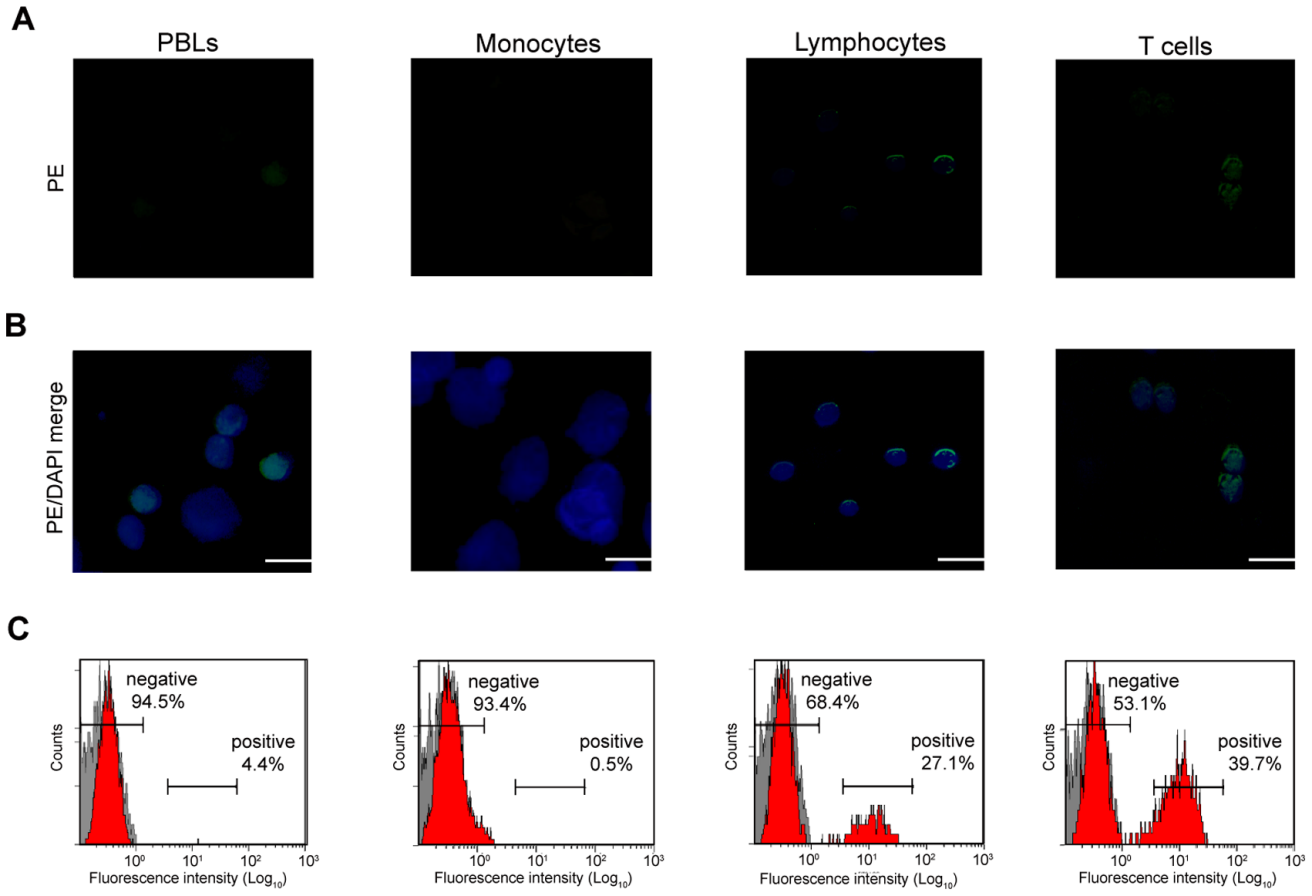
Immunofluorescence stains of *Fugu* CD4^+^ cells in PBLs, monocytes, lymphocytes and T cells. A), B) Cells were reacted with anti-*Fugu* CD4 Ab as primary Ab and goat anti-rabbit IgG (PE) as secondary Ab and DAPI to mark cell nuclei. Scale bar equals 20 µm. C) Cells stained with anti-*Fugu* CD4 Ab were also analyzed by flow cytometry.

PBLs, monocytes, lymphocytes and T cells were characterized by using anti-*Fugu* CD4 Ab. By fluorescence microscopy, the nucleus was stained with DAPI. By indirect immunofluorescence staining with anti-*Fugu* CD4 Ab, followed by PE-conjugated anti rabbit IgG Ab as secondary Ab, it was shown that anti-*Fugu* CD4 Ab reacted on the surface of lymphocytes (Fig. 4A and B); however, monocytes did not show any reactivity to anti-*Fugu* CD4 Ab. Furthermore, in FCM analysis, staining with anti-*Fugu* CD4 Ab revealed that 4.4±1.6% of the PBLs were CD4^+^. In purified monocytes, the reaction with anti-*Fugu* CD4 Ab was low (0.5±0.3%). The ratio of CD4^+^ cells in lymphocytes was 27.1±2.1%. Additionally, the CD4^+^ cells represented a high percentage of T cells (39.7±4.3%) (Fig. 4C). No reaction to each cell population was observed with normal rabbit serum.

**Figure 5:**
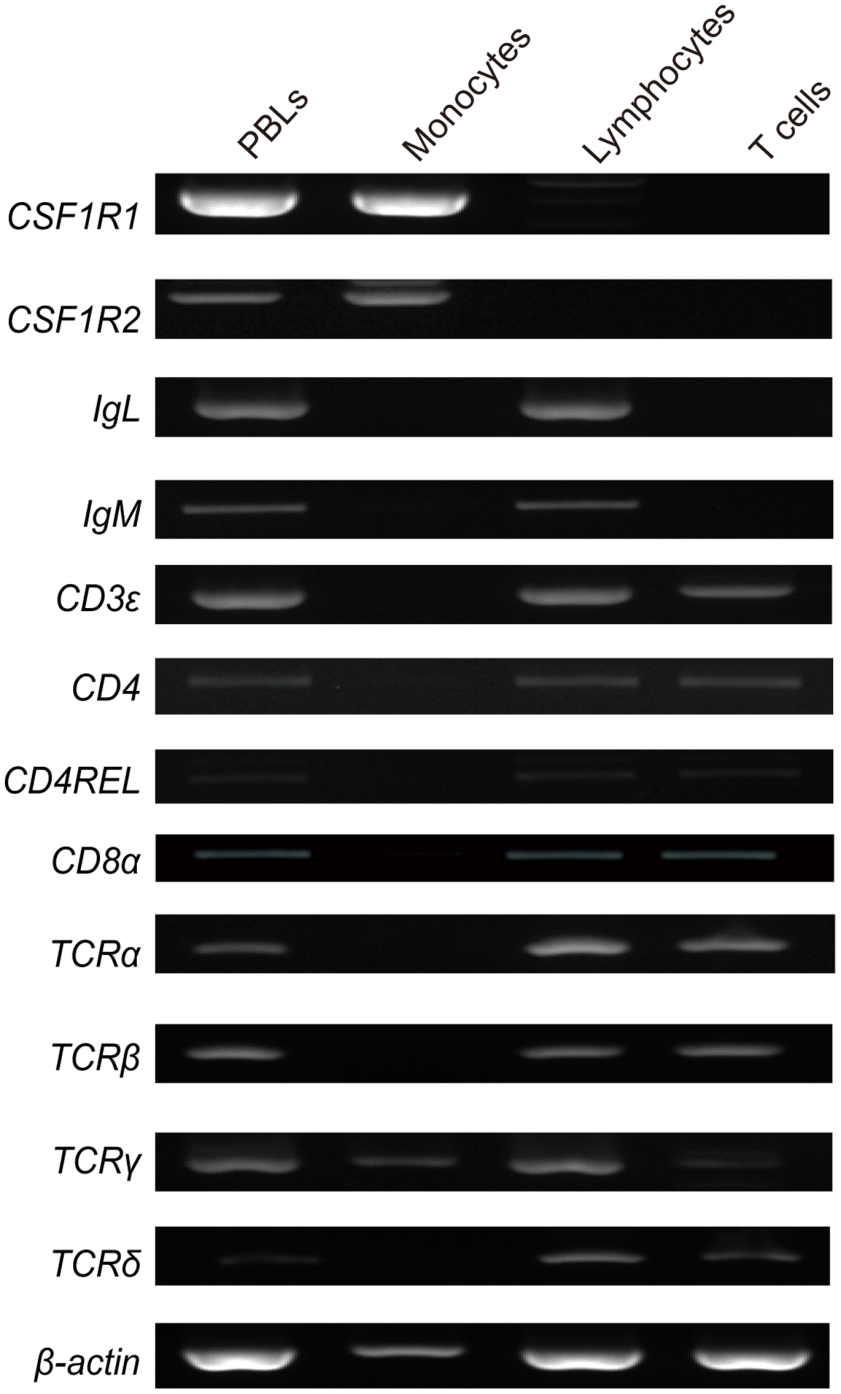
Expression of cell marker genes in *Fugu* PBLs, monocytes, lymphocytes and T cells. The cell marker genes used were a monocyte marker, *CSF1R1*, *-2*, a B-cell marker, *IgL*, *IgM* and T-cell markers, *CD3ε*, *CD4*, *CD4REL*, *CD8α*, *TCRα*, *TCRβ*, *TCRγ* and *TCRδ*.

To identify the cell types of the purified cells from PBLs, the expression patterns of cell marker genes were examined by RT-PCR. The *CSF1R1*, *CSF1R2*, *IgL*, *IgM*, *CD3ε, CD4*, *CD4REL*, *CD8α*, *TCRα, TCRβ, TCRγ and TCRδ* genes were expressed in PBLs. The purified monocytes expressed monocyte marker genes, *CSF1R1* and *CSF1R2*, in addition to a T cell marker gene, *TCRγ*. On the other hand, the purified lymphocytes did not express monocyte marker genes *CSF1R1* and *CSF1R2*. The purified T cells expressed only T-cell marker genes (*CD4*, *CD4REL*, *CD8α*, *CD3ε, TCRα, TCRβ, TCRγ* and *TCRδ*), whereas the B-cell marker gene *IgL*, and *IgM* was not expressed (Fig. 5).

### Characterization of CD4^+^ Cells Isolated by MACS

**Figure 6:**
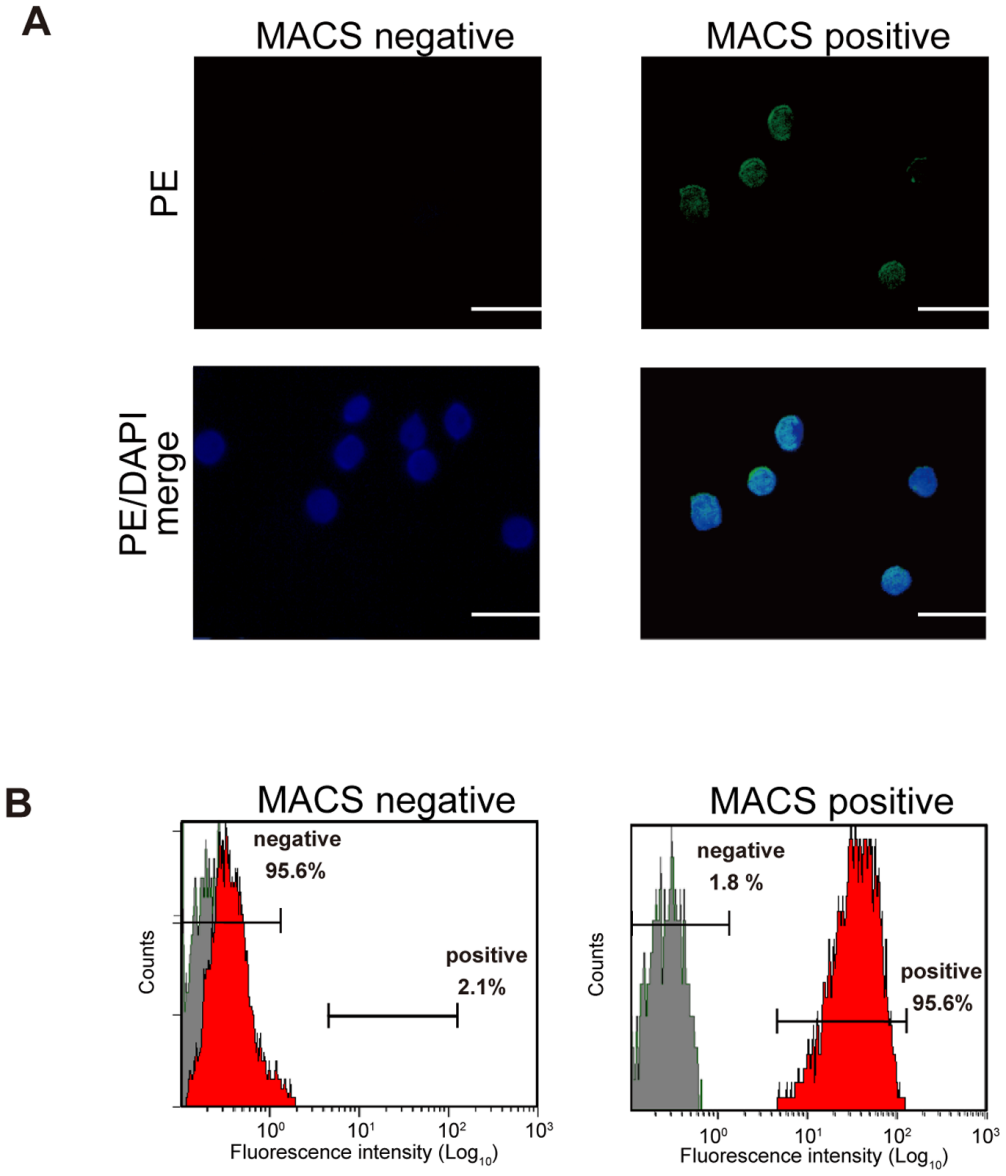
The confirmation of CD4^+^ cells sorting from *Fugu* PBLs by MACS. MACS negative refers to flow through with unlabelled cells. MACS positive refers to elution of positively selected cells. A) Immunofluorescence stains of CD4^+^ cells. B) FCM analysis of CD4^+^ cells.

**Figure 7:**
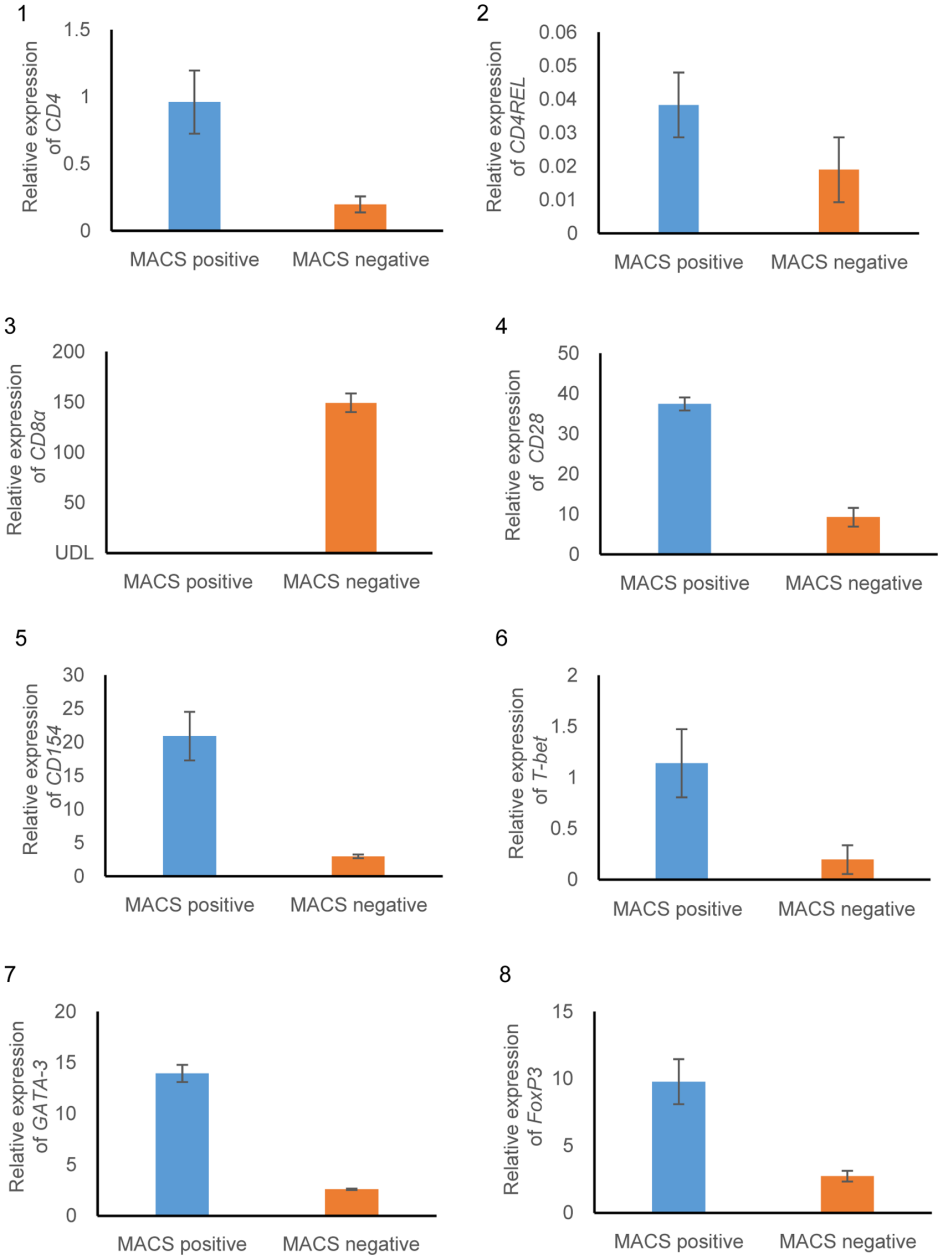
Relative expression levels analyzed by real-time PCR. The comparative threshold cycle (Ct) method was used to determine relative transcript levels, using β-actin as a housekeeping control. The analysed genes were as follows: 1) *CD4*, 2) *CDREL*, 3) *CD8α*, 4) *CD28*, 5) *CD 154*, 6) *T-bet*, 7) *GATA-3* and 8) *FoxP3*. Data are presented as mean ± S.D. of triplicate samples.

We isolated CD4^+^ cells from peripheral blood T cells by using MACS. In an immunofluorescence analysis of the isolated magnetically positive or negative cells, most MACS-positive cells showed reactivity to anti-*Fugu* CD4 Ab. On the other hand, the anti-*Fugu* CD4 Ab scarcely reacted with MACS-negative cells (Fig. 6A). The percentage of MACS-positive cells reacting with the anti-*Fugu* CD4 Ab was about 95.6±3.1% by the FCM analysis, whereas only 2.1±1.1% of MACS-negative cells reacted to the Ab (Fig. 6B). Relative expression analysis of lymphocyte markers was used for further phenotypic identification. As shown in Fig. 7, *CD4* and *CD4REL* genes were highly expressed in MACS-positive cells compared with that of MACS negative cells. *CD154,* a marker for activated CD4^+^ T lymphocytes was expressed higher in MACS-positive cells than in MACS-negative cells. *CD28,* a T cell marker was expressed in both MACS-positive and negative cells. *CD8α* gene was detected in MACS-negative cells but was under detection limit (UDL) in MACS-positive cells. In addition, transcriptional factors related to Th differentiation (*T-bet*, *GATA-3* and *Foxp3* genes) were expressed higher in MACS-positive cells than in MACS-negative cells.

### Multiplex RT-PCR Assay of Cytokine Genes Expressed in *Fugu* CD4^+^ Cells

**Figure 8:**
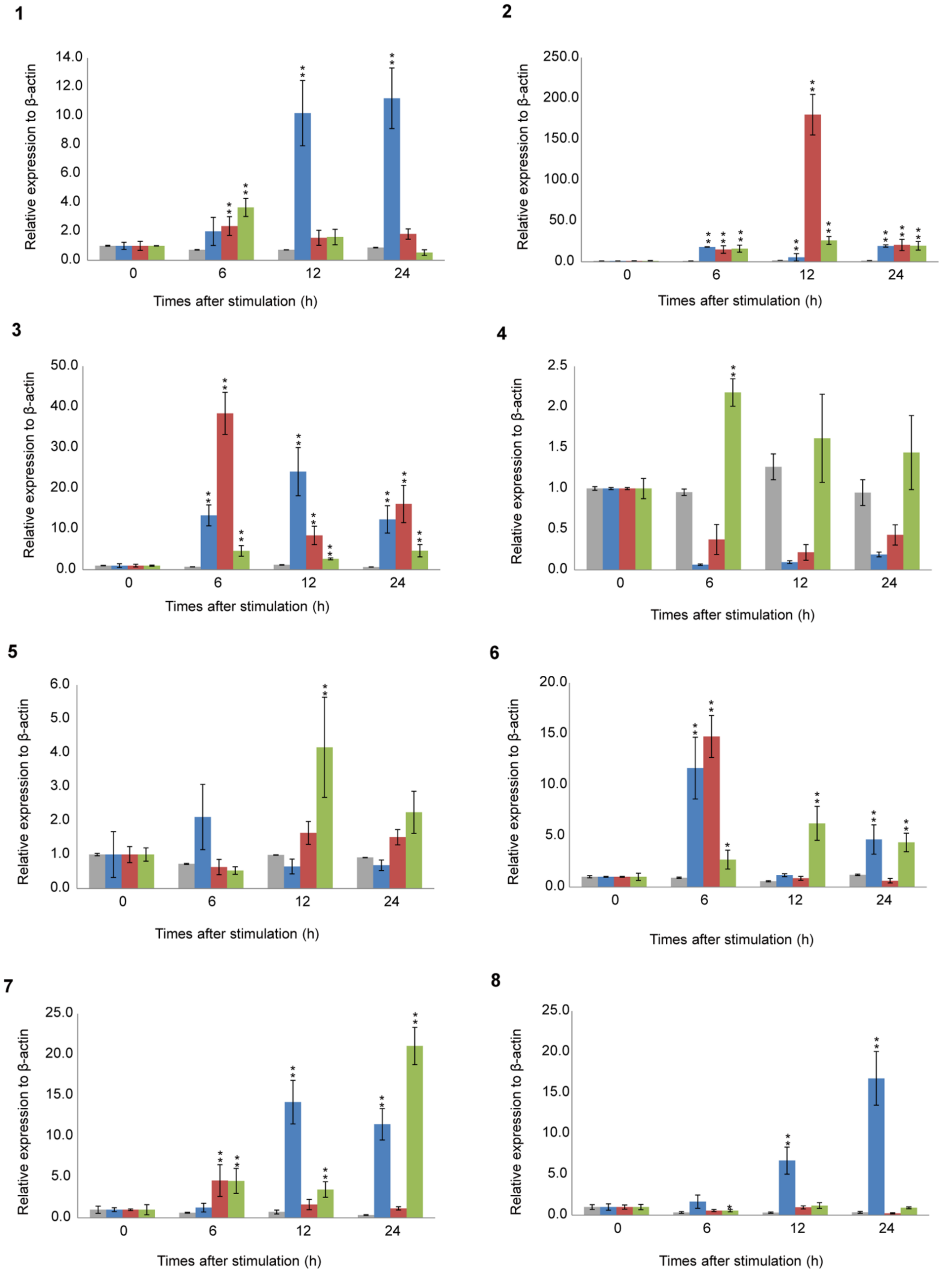
Expression analysis of *Fugu* cytokine genes in CD4^+^ cells sorted from PBLs under immunostimulatory conditions. RNA isolated from CD4^+^ cells incubated with 20 µg/ml LPS, 20 µg/ml polyI:C or 20 µg/ml ConA for 0 (cont.), 6, 12 and 24 h. *Fugu* cytokine mRNA transcripts were determined by multiplex RT-PCR and standardized to the respective β-actin mRNA. The analysed genes were as follows: 1) *IL-2*, 2) *IFN-γ,* 3) *TNF-α*, 4) *IL-4/13A*, 5) *IL-4/13B*, 6) *IL-17A/F-3*, 7) *TGF-β1*, and 8) *IL-10*. Data are presented as mean ± S.D. of triplicate samples. *P<0.01 as compared to the control (upregulation). The relative expression level is expressed as arbitrary units where one unit is equal to the average expression level of each cytokine gene in CD4^+^ cells from unstimulated PBLs. Gray, Blue, red and green bars indicate HBSS, LPS, polyI:C and ConA-stimulations, respectively.

To further characterize the phenotype of *Fugu* CD4^+^ cells sorted from PBLs under immunostimulatory conditions, the cytokine gene expression profile was analyzed by multiplex RT-PCR. The assay determines the relative change of cytokine gene expression in CD4^+^ cells after LPS, polyI:C or ConA stimulation. The expression level of some cytokine genes was high in stimulated CD4^+^ cells but low in unstimulated CD4^+^ cells (Fig. 8 and Table S1).

*Fugu IL-2*, *IFN-γ* and *TNFα* which correspond to Th1 cytokines in mammals, were significantly increased following stimulation with LPS, polyI:C and ConA as compared to the control. The *IFN-γ* gene was increased more than 180-fold in CD4^+^ cells at 12 h post polyI:C stimulation as compared to the control (*P*<0.01; Fig. 8). More than a 5-fold increase in *IFN-γ* expression was detected in the LPS stimulation group (Fig. 8). In the ConA group, the greatest increase in *IFN-γ* gene expression occurred at 12 h (Fig. 8). The expression of the *IL-2* gene was also significantly increased at 12 h and 24 h post LPS stimulation as compared to the control (*P*<0.01; Fig. 8). However, the *IL-2* gene response after treatment with polyI:C was not significant as compared to the control at 12 h and 24 h (Fig. 8). *IL-2* gene expression increased about 4-fold relative to the control at 6 h after ConA stimulation (*P*<0.01; Fig. 8). A significant increase in *TNFα* gene expression as compared to the control occurred 12 h after LPS stimulation (24-fold, *P*<0.01; Fig. 8). Stimulation with polyI:C led to increased expression of the *TNFα* gene up to 38-fold at 6 h after stimulation. ConA stimulation up-regulated the *TNFα* gene at 6 h post stimulation as compared to the control (4.6-fold, *P*<0.01; Fig. 8).

Th2 cytokines (*IL-4/13A* and *-B* genes) were not increased in CD4^+^ cells stimulated with LPS or polyI:C (Fig. 8). The *IL-4/13A* gene was markedly decreased as compared with the control after LPS and polyI:C stimulation. On the other hand, ConA stimulation slightly increased the expression of *IL-4/13A* (2.2-fold, *P*<0.01 at 6 h) and *IL-4/13B* (4.6-fold, *P*<0.01 at 12 h; Fig. 8).

The Th17 cytokine gene, *IL-17A/F-3*, showed a significant increase in expression at 6 h post LPS stimulation as compared to the control (about 10-fold, *P*<0.01; Fig. 8). PolyI:C caused an 15-fold increase in *IL-17A/F-3* after 6 h (*P*<0.01) that decreased at later time points. ConA caused a 6.2-fold increase in *IL-17A/F-3* expression as compared to the control at 12 h post stimulation.

In addition to the Th1, Th2 and Th17 cytokines, the *TGF-β1* and *IL-10* genes expressed by Treg cells were examined. *TGF-β1* showed an increase in expression 6 h after polyI:C stimulation, whereas the increase following ConA stimulation gradually increased from 6 h post stimulation with the highest expression occurring after 24 h (Fig. 8). LPS stimulation elevated the expression of *TGF-β1* over 10-fold as compared to the control at 12 h and 24 h (*P*<0.01; Fig. 8). The *IL-10* gene (also a Th2 cytokine) was gradually increased by LPS treatment as compared to the control (*P*<0.01; Fig. 8), and the greatest increase occurred 24 h post stimulation. PolyI:C yielded almost the lowest expression level at any time point after stimulation as compared to the control (Fig. 8). The highest expression of the *IL-10* gene was 2.3-fold at 6 h post stimulation with ConA, and the expression gradually decreased at later time points.

## Discussion

The present study describes the production of a specific anti-*Fugu* CD4 Ab and the isolation of CD4^+^ cells from pufferfish PBLs by MACS. Although in recent years the identification and characterization of cell-surface markers specific to T cells has rapidly increased in teleosts, there are few antibodies against these markers [14]. The produced anti-*Fugu* CD4 Ab reacted with recombinant *Fugu* CD4 and lysates of CHO cells expressing *Fugu* CD4 (*Fugu* CD4^+^-CHO cells) and *Fugu* PBLs. Moreover, in the FCM analysis, the detection level of CHO-*Fugu* CD4 cells overexpressing the *Fugu* CD4 protein on the cell surface was high. Currently, some antibodies have been developed to recognize fish T cells, B cells, and common leucocyte surface antigens [14]. Among them, an anti-*Fugu* CD8 antibody showed reactivity to CD8 protein derived from PBL lysates and allowed the identification of CD8 protein on the cell surface [44]. In another fish, an antibody (6D1) to ginbuna CD4 was produced and has allowed the identification of CD4^+^ cells by FCM and immunofluorescense, however it did not detect the CD4 protein in PBL lysates by Western blotting [15]. The antibody to CD4 in green pufferfish detected CD4 in spleen and head kidney by FCM analysis [16]. These reports suggest that antibodies generated to cell surface proteins show different reactivities depending on the status of the sample (live cells, fixed cells, or cell lysate) or detection method. The anti-*Fugu* CD4 Ab produced in the present study shows good reactivity to both the native form of CD4 expressed on the surface of live cells and the denatured form of CD4 transferred to a membrane and used in Western blotting.

For the efficient sorting of CD4^+^ cells from *Fugu*, we previously purified T cells from PBLs. The cell markers expressed in monocytes, lymphocytes and T cells analysed in this study were previously reported [40], [45], [46], and each marker gene was expressed only in a specific cell type. Moreover, analysis of morphology and FCM of blood leukocytes have been reported in several fishes [47], [48], [49]. The reports reveal that fish leukocytes possess similar characteristics to mammalian leukocytes. Therefore, the results of this study are consistent with previous reports and confirm the successful purification of T cells from PBLs. However, the T-cell marker gene *TCRγ* was detected in the purified monocytes, and FCM analysis indicated a slight population in the lymphocyte gate. These results suggest a low level of contamination of lymphocytes. The FCM analysis of PBLs with anti-*Fugu* CD4 Ab revealed the percentage of CD4^+^ cells to be 4.4%. Toda *et al*. [15] reported that 14% of CD4^+^ cells were present in ginbuna PBLs. Accordingly, it was suggested that the difference in the percentage of CD4^+^ cells may be attributed to differences in the fish species, maturation and immune status.

By using T cells purified from PBLs, we sorted the cells expressing CD4 on the cell surface with MACS. *CD4*, *CD28* and *CD154* genes were expressed in both MACS-positive and negative cells, indicating that some CD4^+^ cells did not bind to the MACS column. And, CD8α was expressed only in MACS negative cells. These results supported the characterization of MACS-positive cells by FCM and immunofluorescence analysis. The sorted CD4^+^ cells expressed two CD4 molecule genes (Fig. 7). However, the peptide sequence of the antigen used for the production of anti-CD4 Ab was not present in CD4REL. Moreover, no reaction of the Ab to the synthesized rCD4REL (Fig. S2) and the CD4REL (approx. 34 kDa; Fig. 1-D) in *Fugu* PBLs was confirmed in Western blotting. Therefore, the CD4 cells sorted in this study are likely CD4, CD4REL double positive or CD4 single positive cells. It has been reported that an identical expression profile in trout strongly suggested the co-expression of *CD4* and *CD4REL* in T cells [30]. Catfish *CD4* was not expressed in enriched IgM+ B cells, but *CD4REL* was expressed [32]. However, both trout *CD4* and *CD4REL* genes were not expressed in sIgM^+^ lymphocytes [30]. Toda *et al.* [15] reported that CD4 sorted cells co-expressed the *CD4REL* gene in ginbuna. Wen *et al.* [16] reported that *Tetraodon* CD4REL^+^ cells, rather than CD4^+^ cells, showed binding capacity with both MHC-II and IL-16 and was largely responsible. Taken together, we hypothesize that teleosts possess CD4 or CD4REL single positive and CD4, CD4REL double positive T cells. However, to date, the identification of CD4^+^ subset cells and their functions has been limited. It is unclear whether these CD4^+^ cells in fish are equivalent to a particular kind of effector cell in mammals. Therefore, the expression analysis of specific transcription factors for each effector cell was conducted.

The sorted *Fugu* CD4^+^ cells expressed *T-bet*, *GATA-3* and *Foxp3*, which are master transcription factors of Th1, Th2, and Treg cell differentiation, respectively [50]. Takizawa *et al.* [51] reported that ginbuna *T-bet* was strongly expressed in IgM-negative lymphocytes in comparison with IgM-positive lymphocytes. These results indicate that T-bet might play an important role in regulating the Th1 response in fish. High expression levels of salmonid *GATA-3* and Th2 hallmark cytokine *IL-4/13A* genes have been found in the thymus, skin and gill [52]. The tissue distribution may associate GATA-3 with a Th2-like response in mammals [52]. Foxp3, an important transcription factor in Treg cells, was detected in CD4-2^+^CD25-like^+^ cells, but not in CD4-2^+^CD25-like^−^ cells in *Tetraodon* [16]. These reports indicate that teleost T-bet, GATA-3 and Foxp3 might regulate the Th subset development. Accordingly, the sorted *Fugu* CD4^+^ cells in this study should contain the Th and Treg population.

Several distinct types of Th cells including Th1, Th2, Th17 and Treg cells are presumed to be the progeny of naive CD4^+^ T cells responding to specific antigen [53], [54]. Therefore, to analyse whether fish CD4^+^ cells express Th specific cytokines, the CD4^+^ cells were sorted from PBLs stimulated with LPS, polyI:C or ConA, and the expression profile of cytokine genes in these cells was analysed. In mammals, several studies reported that LPS or polyI:C-stimulated APC promotes a Th1 profile of cytokine secretion in CD4^+^ T cells [55]. In this study, PBLs including several cells like B cells and monocytes were stimulated with LPS or polyI:C, and these antigen-presenting cells in leukocytes might induce the expression of specific Th1 cytokines, especially *IFN-γ* and *TNFα*, in CD4^+^ cells. Moreover, the expression of *Fugu IL-4/13A, IL-4/13B* genes was decreased by polyI:C and LPS stimulation, whereas Th1 cytokines like *IFN-γ*, showed high expression. In mammals, a shift from a Th1 to a Th2 cytokine expression profile was observed in polyI:C stimulation. Furthermore, it is generally believed that LPS prevents a Th2 reaction by inducing a Th1 response to switch the balance of Th1/Th2 immunity [56]. In fish, Takizawa *et al*. [52] described a Th2 cytokine, trout *IL-4/13A*, that lacked sensitivity to polyI:C in *in vitro* studies and differed in expression patterns compared with the Th1 cytokine gene *IFN-γ*. Additionally, T-cell enriched *Fugu* PBLs expressed more *IL-4/13A* and *IL-4/13B* after stimulation with recombinant B7 molecules, while *IFN-γ* expression was decreased [40]. Therefore, our results and previous reports strongly suggest that Th1 dominance inhibits differentiation of naive Th0 cells into Th2 cells in fish as well as in mammals. However, in this study, we were unable to induce a Th2 response *in vitro*. Therefore, to classify the paradigm of Th1 and Th2 in fish, a parasite infection is required to induce the Th2 lineage.

Moreover, we analysed the expression of Th17 cytokine, *IL-17A/F-3*. In this study, the *IL-17A/F-3* gene was upregulated at an early stage (6h) after stimulation with LPS and polyI:C in immunostimulated CD4^+^ cells. In lamprey, the VLRA lymphocytes (T-like lymphocytes) respond to LPS and upregulate the expression of the *IL-17* gene [57]. Similarly to this report, it was reported that the expression of the fish *IL-17* gene was upregulated with LPS [58]. In addition, in mammals IL*-*17 has been shown to be an important mediator for inflammatory responses [59]. Taken together, the increase of *IL-17A/F-3* gene expression may suggest an increase of Th17 cytokine expression by LPS stimulation. In addition, the upregulation of *IL-17A/F-3* gene expression by polyI:C stimulation may suggest the regulation of the inflammatory response.

In mammals, Treg cytokines TGFβ and IL-10 suppress the function of other Th subsets to limit the immune response, and the suppressor mechanism of Treg cells acts on antigen presenting cells like dendritic cells [60], [61]. We observed the strong upregulation of *TGFβ1* and *IL-10* genes after treatment with LPS but not polyI:C. A mechanism of feedback regulation, in which IL-10 and TGFβ1 were upregulated in a pro-inflammatory stimulation, was confirmed [41], [62]. In this study, it was suggested that Treg cells express suppressive cytokines by stimulation of LPS as an inflammatory inducer. However, IL-10 was recently confirmed to be produced by not only Treg cells, but also Th2 and a novel Th subset, Th9, in mammals [63]. For further analysis, it is necessary to confirm the phenotype of IL-10-producing CD4^+^ cells by determining the specific cell-surface markers in these cells and isolating these cells with antibody against the specific markers. The suppressive regulation of fish immunity can be further clarified by such studies.

ConA is a lectin that non-specifically activates T cells [64]. Moreover, ConA treatment was suggested to non-specifically activate all Th cells, unlike LPS and polyI:C, and up-regulate the expression of different Th cytokines [65]. CD4^+^ cells stimulated with ConA can induce differentiation to effector cells [66]. In this study, non-specific mitogenic responses following ConA treatment upregulated Th1, Th2, Th17 and Treg cytokines in *Fugu* CD4^+^ cells. These results showed that fish CD4^+^ cells were up-regulated by ConA treatment, similar to the process in mammals.

In conclusion, a combination of methods involving density gradient centrifugation, cell adhesion and magnetic cell sorting made it possible to obtain CD4^+^ cells from *Fugu* blood. The expression profile of cytokine genes determined by a multiplex RT-PCR assay revealed that several distinct Th cytokines were expressed in CD4^+^ T cells. Our results suggest that Th subsets exist in fish and that the orientation of immune responses is regulated by Th cytokines expressed from the cells, as occurs in mammals. These results provide the first evidence of cytokine dynamics and their correlation in fish CD4^+^ cells. To further characterize Th cells in fish, we need to characterize the expression profile (at the gene and protein levels) of cytokines in CD4^+^ cells (including CD4REL^+^ cells) under immunostimulatory conditions or isolated from fish infected with pathogens. In addition, the identification and characterization of CD4^+^ cells except for T cells will be needed for fish immunology field.

## Supporting Information

Figure S1 **Reactivity of anti-** ***Fugu*** **CD4 Ab against synthesized CD4 peptide in ELISA.** Triangle △) and circle ○) indicate anti-*Fugu* CD4 Ab and rabbit normal serum used as a control, respectively.
(TIF)

Figure S2 **The confirmation of antibody specificity using Western blotting.** The recombinant *Fugu* CD4REL, FLAG-tagged was detected with A) anti-FLAG Ab, but not B) anti-*Fugu* CD4 Ab. The position of molecular weight markers is indicated to the left of each Western blot. The arrow indicates the predicted size of *Fugu* CD4REL protein.
(TIF)

Table S1 (XLSX)
