# Supplementary figures and images for: Cytokine Gene Expression in CD4 Positive Cells of the Japanese Pufferfish, Takifugu rubripes
Source: PLoS One. 2013 Jun 18;8(6):e66364. doi: 10.1371/journal.pone.0066364 (PMC3688880; doi:10.1371/journal.pone.0066364)

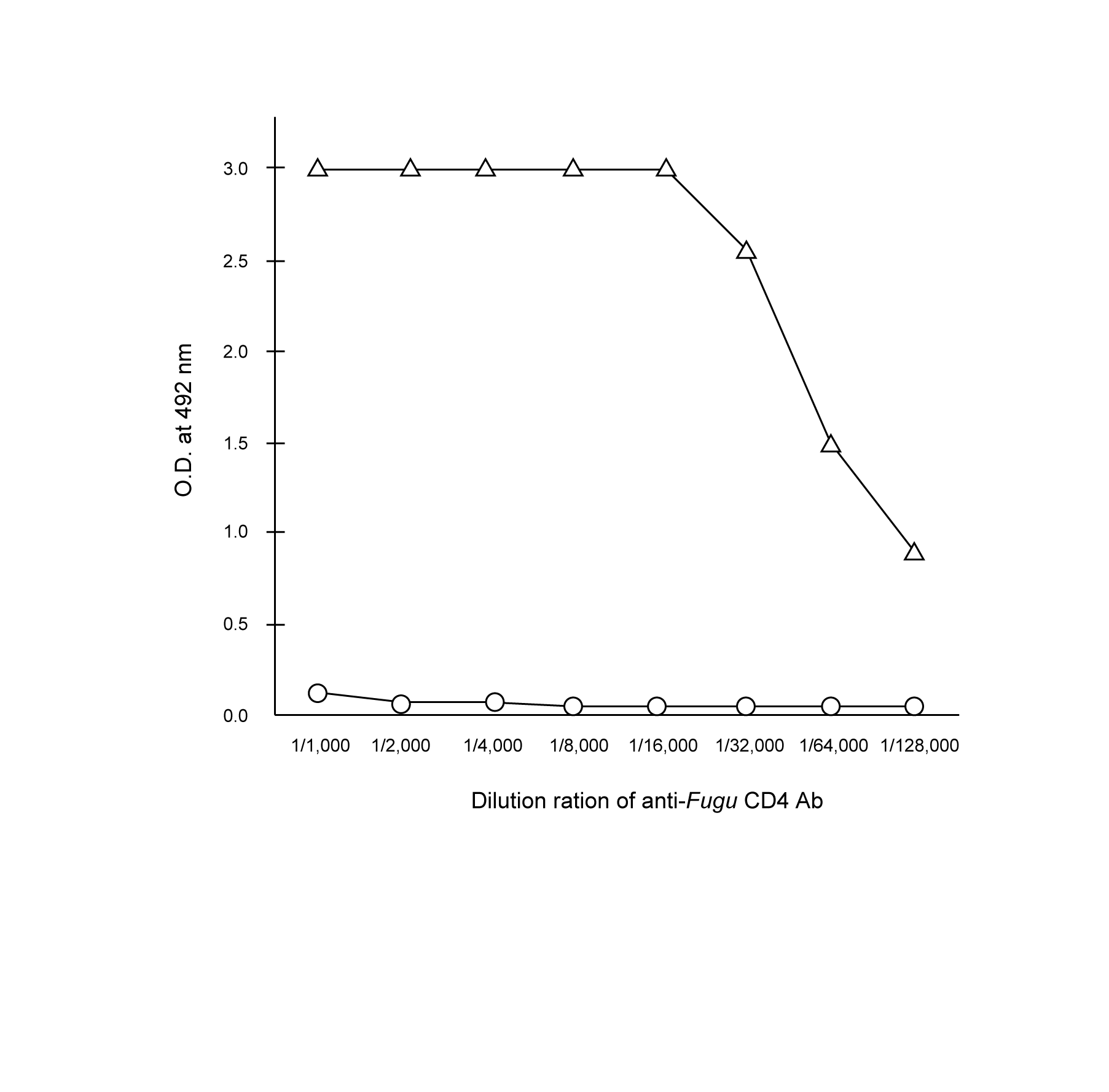

Supplement: Figure S1 — Reactivity of anti- Fugu CD4 Ab against synthesized CD4 peptide in ELISA. Triangle △) and circle ○) indicate anti-Fugu CD4 Ab and rabbit normal serum used as a control, respectively. (TIF) [file pone.0066364.s001.tif]

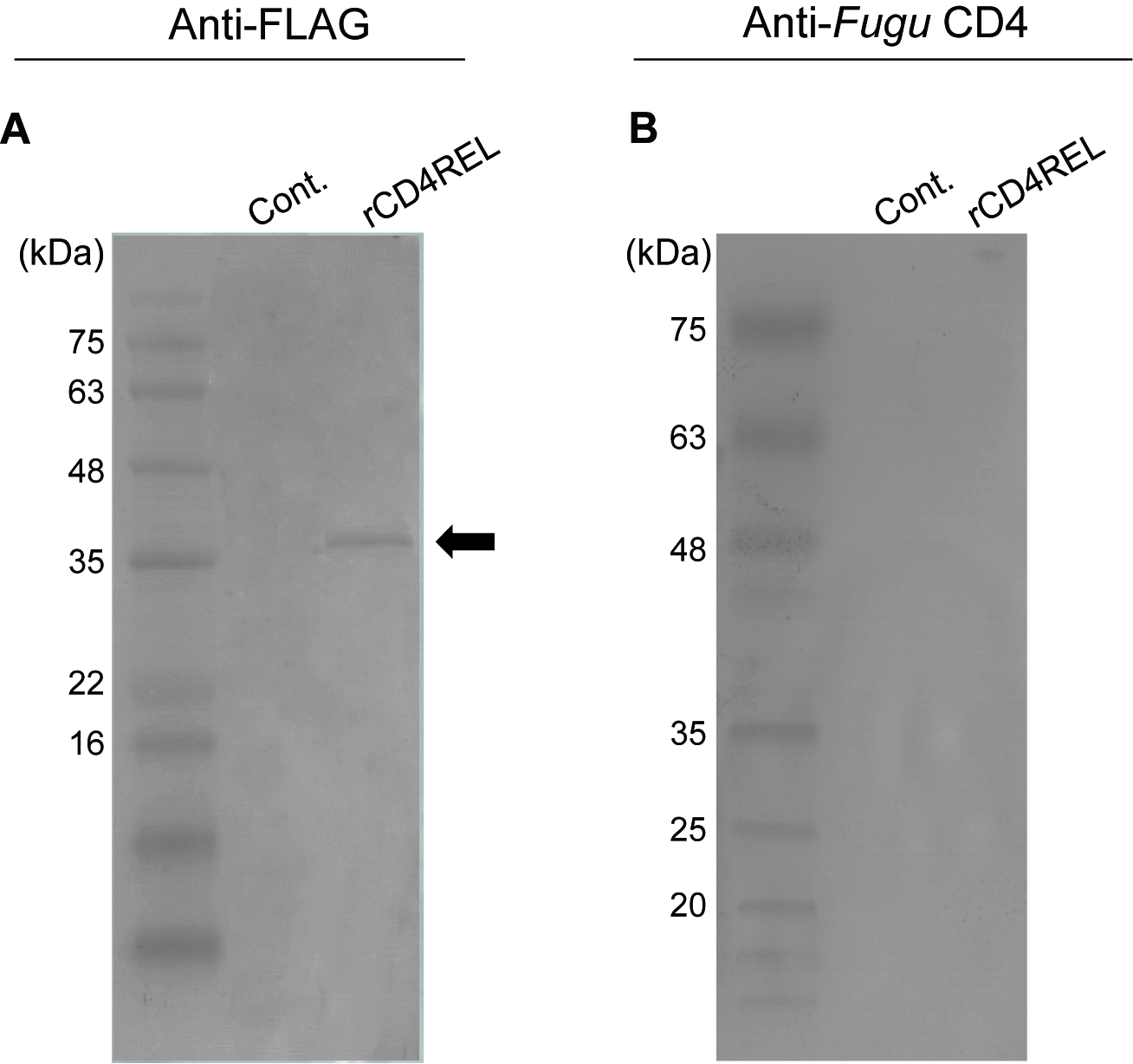

Supplement: Figure S2 — The confirmation of antibody specificity using Western blotting. The recombinant Fugu CD4REL, FLAG-tagged was detected with A) anti-FLAG Ab, but not B) anti-Fugu CD4 Ab. The position of molecular weight markers is indicated to the left of each Western blot. The arrow indicates the predicted size of Fugu CD4REL protein. (TIF) [file pone.0066364.s002.tif]
